# Progress Report of the Tohoku Medical Megabank Community-based Cohort Study: Study Profile of the Repeated Center-based Survey During Second Period in Miyagi Prefecture

**DOI:** 10.2188/jea.JE20230241

**Published:** 2024-09-05

**Authors:** Atsushi Hozawa, Kumi Nakaya, Naoki Nakaya, Tomohiro Nakamura, Mana Kogure, Rieko Hatanaka, Ippei Chiba, Ikumi Kanno, Junichi Sugawara, Eiichi Kodama, Yohei Hamanaka, Tomoko Kobayashi, Akira Uruno, Naho Tsuchiya, Takumi Hirata, Akira Narita, Akito Tsuboi, Toru Tamahara, Akihito Otsuki, Maki Goto, Makiko Taira, Ritsuko Shimizu, Kichiya Suzuki, Taku Obara, Masahiro Kikuya, Hirohito Metoki, Mami Ishikuro, Inaho Danjoh, Soichi Ogishima, Satoshi Nagaie, Naoko Minegishi, Masahiro Hiratsuka, Kazuki Kumada, Ichiko Nishijima, Takahiro Nobukuni, Yumi Yamaguchi-Kabata, Fuji Nagami, Shigeo Kure, Nobuo Fuse, Kengo Kinoshita, Yoko Izumi, Shinichi Kuriyama, Masayuki Yamamoto

**Affiliations:** 1 Tohoku Medical Megabank Organization, Tohoku University, Sendai, Japan; 2 Graduate School of Medicine, Tohoku University, Sendai, Japan; 3 Faculty of Data Science, Kyoto Women’s University, Kyoto, Japan; 4 Suzuki Memorial Hospital, Sendai, Japan; 5 Yamato Home Medical Care Clinic Kurihara, Miyagi, Japan; 6 Institute for Clinical and Translational Science, Nara Medical University Hospital, Nara, Japan; 7 Department of Hygiene and Public Health, Teikyo University School of Medicine, Tokyo, Japan; 8 Division of Public Health, Hygiene and Epidemiology, Faculty of Medicine, Tohoku Medical and Pharmaceutical University, Sendai, Japan; 9 Miyagi Children’s Hospital, Sendai, Japan; 10 Graduate School of Information Sciences, Tohoku University, Sendai, Japan; 11 Institute of Development, Aging, and Cancer, Tohoku University, Sendai, Japan; 12 International Research Institute of Disaster Science, Tohoku University, Sendai, Japan

**Keywords:** prospective cohort studies, Great East Japan Earthquake, genome cohort

## Abstract

**Background:**

The purpose of this study was to report the basic profile of the Miyagi Prefecture part of a repeated center-based survey during the second period of the Tohoku Medical Megabank Community-Based Cohort Study (TMM CommCohort Study), as well as the participants’ characteristics based on their participation type in the baseline survey.

**Methods:**

The second period survey, conducted from June 2017 to March 2021, included participants of the TMM CommCohort Study (May 2013 to March 2016). In addition to the questionnaire, blood, urine, and physiological function tests were performed during the second period survey. There were three main ways of participation in the baseline survey: Type 1, Type 1 additional, or Type 2 survey. The second period survey was conducted in the same manner as the Type 2 survey, which was based on the community support center (CSC).

**Results:**

In Miyagi Prefecture, 29,383 (57.7%) of 50,967 participants participated in the second period survey. The participation rate among individuals who had visited the CSC was approximately 80%. Although some factors differed depending on the participation type in the baseline survey, the second period survey respondents in the Type 1 and Type 2 survey groups at baseline had similar traits.

**Conclusion:**

The second period survey of the TMM CommCohort Study provided detailed follow-up information. Following up on the health conditions of the participants will clarify the long-term effects of disasters and contribute to personalized prevention.

## INTRODUCTION

The Tohoku Medical Megabank (TMM) Project, a large-scale genomic cohort research project centered on the Great East Japan Earthquake (GEJE) disaster area, aims to contribute to the reconstruction of medical care and medical institutions in the area and the development of a next-generation medical system that includes drug discovery and personalized medicine.^1^ The TMM project is conducted in Miyagi and Iwate Prefectures, with the TMM Birth and Three-Generation Cohort Study (The TMM BirThree Cohort Study),^2^ which is a birth and three-generation cohort study, and the TMM Community-Based Cohort Study (The TMM CommCohort Study), which is a population-based adult cohort study.^3^

The purpose of the TMM CommCohort Study is to investigate the influence of earthquake-related disaster on health, support local people by providing avenues for the early detection of illness, and provide individualized treatment.^3^ To date, various results from the baseline survey have been published.^4^^–^^11^ The Miyagi part of the baseline survey of the TMM CommCohort Study was conducted from May 2013 to March 2016, approximately 2 years after the GEJE. The second period survey was conducted approximately 4 years subsequently, from June 2017 to March 2021. The third phase, a detailed center-based repeat survey, started in July 2021 is scheduled to end in March 2026.

There were three main ways to participate in the baseline survey: Participation in the Type 1, Type 1 additional, or Type 2 survey (Table 1).^3^ Participant characteristics at baseline differed depending on the participation type.^3^ Details of the differences have been described in a previous paper.^3^ The second period survey employed the same recruiting method as the Type 2 survey (ie, all participants were asked to visit one of the community support centers [CSC] for a detailed examination). Visiting the CSC allowed participants to undergo detailed physiological functional tests. Therefore, in this profile paper, we investigated the classification of Type 1 without CSC survey, Type 1 with CSC survey, and Type 2 survey since the characteristics of participants at baseline might differ based on whether they visited the CSC or not (Table 1).^3^

**Table 1.** Baseline survey participation type

Based on the baseline survey profile paper^3^	The way of participation	Based on this second period survey profile paper	The way of participation
**Type 1 survey**	Recruited participants at the specific health check-up venues of 28 municipalities.	**Type 1 without CSC survey**	Type 1 survey or Type 1 additional survey participants **without** visiting the CSC.
**Type 1 additional survey**	Conducted on different dates from those of the specific health check-up in the municipality because of a limitation with the settings (the size of the venue).	**Type 1 with CSC survey**	Type 1 survey or Type 1 additional survey participants **with** visiting the CSC. Participants voluntarily visited the CSC, after participating in a Type 1 survey or Type 1 additional survey.
**Type 2 survey**	Performed at the CSC, which was established by ToMMo in seven areas in Miyagi prefecture. Participants voluntarily visited the CSC.	**Type 2 survey**	Same as the baseline profile paper

CSC, Community Support Centers (Established by ToMMo in seven areas in Miyagi Prefecture).

This study reports the recruitment method, survey variables, and outline of the respondents in the second period survey of the Miyagi Prefecture part of the TMM CommCohort Study. We also compared the baseline characteristics of those who participated in the second period survey with those of participants who did not. Furthermore, the basic characteristics of the participants in the second period survey are shown based on the baseline participation type.

## METHODS

### Recruitment

We described the details of the TMM CommCohort baseline survey.^3^ Briefly, the baseline survey was conducted from May 2013 to March 2016, targeting residents of Miyagi Prefecture aged 20 years or older. Miyagi Prefecture suffered great damage from the GEJE that struck on March 11, 2011. The Type 1 survey was performed at specific municipal health checkup sites, and the Type 2 survey was performed at the CSC, which was established by Tohoku University Tohoku Medical Megabank Organization (ToMMo) in seven areas in Miyagi Prefecture (Table 1). Type 2 participants voluntarily visited the CSC. The Type 1 additional survey was conducted on different dates from those of the specific health check-up in the municipality because of a limitation with the settings (the size of the venue). Among Type 1 participation type (Type 1 survey and Type 1 additional survey), the group that did not visit the CSC later was referred to as Type 1 without CSC survey, while the group that visited was referred to as Type 1 with CSC survey (Table 1). Visiting the CSC allowed participants to undergo detailed physiological functional tests that were not performed in the Type 1 without CSC survey. In this study, we investigated the classification of Type 1 without CSC survey, Type 1 with CSC survey, and Type 2 survey because the characteristics of participants at baseline may alter depending on whether or not they visited the CSC.^3^

The second period survey was conducted by mailing pamphlets to baseline survey participants in the order in which they participated in the baseline survey. Those who wished to participate made reservations via telephone. The secretariat mailed the questionnaire and urine collection kit to the applicant in advance and collected them on the day of participation in the second period survey.

Some participants who could not visit the CSC, mainly due to the spread of coronavirus disease 2019 or poor health, were asked to return only the filled questionnaire. This study examined participants who submitted the questionnaire and blood samples.

After receiving sufficient explanation regarding the study, all participants gave their written informed consent. This study was approved by the Ethical Committee of ToMMo (the first approval: 2012-4-617 and latest approval: 2023-4-004).

### Variables

#### Questionnaire

In addition to basic information such as height, weight, current residence, work, income, family background, medications, and personal and family medical histories, we asked about nursing care status, memory at the time of the earthquake and disaster prevention efforts, social isolation measures (Lubben Social Network Scale [LSNS-6]),^12^ irritable bowel syndrome (ROME-II modular questionnaire),^13^^,^^14^ food intake (Food Frequency Questionnaire), and eating habits. In addition, on the day of the survey, participants answered questions about physical activity, smoking status, drinking status, depressive symptoms (Center for Epidemiologic Studies Depression Scale [CES-D]),^15^ psychological distress (Kessler Psychological Distress Scale [K6]),^16^ and insomnia (Athens Insomnia Scale [AIS]),^17^ on a tablet (Table 2).

**Table 2.** Details of the questionnaire and tablet survey at baseline and/or follow-up for the TMM CommCohort study

Measurement	Measurement lists	Baseline	second period
Basic information	Sex (C)	○	
	Birthday and age (N)	○	
	Body weight/Body height (N)	○	○
	Body weight at 20 years old (N)	○	
	Birth weight (C)	○	
	Education (C)	○	
	Degree of house damage from the disaster (C)	○	
	Current residence (C)	○	○
	Number of relocations following the GEJE (C)	○	○
	Income (C)		○
Daily activity	Daily activity (C)	○	○
	Transportation (C)		○
	Surrounding environment (park/store) (C)		○
Drinking	Drinking status (C)	○	○
	Drinking frequency (C)	○	○
	Amount of drinking (C)	○	○
Smoking	Smoking status (C)	○	○
	Reasons for quitting smoking (C)	○	○
	Passive smoking (C)	○	○
Stress	Perceived stress (C)	○	○
	Psychological distress (Kessler Psychological Distress Scale [K6]) (C) (N)	○	○
	PTSD scale (Impact of Event Scale-Revised [IES-R])	○	
	PTSD (C)		○
	Holmes-Rahe Social Readjustment Rating Scale (C)	○	
Drug, supplements, and health foods	Use of drug, supplements, and health foods (C)	○	○
Family	Marital status (C)	○	○
	Number of children (N)	○	○
	Cohabitants (C)	○	○
	Caring for the family (C)		○
Health status	Current health status (C)	○	○
	Treatment of hypertension, hyperlipidemia and diabetes mellites (C)	○	○
	Medical history (C)	○	○
	Dental consultation (C)	○	○
	Helicobacter pylori infection/eradication (C)		○
Working status	Employment status (C)	○	○
	Profession (C)	○	
	Engaged industry (C)	○	○
Sleeping	Sleeping time (C)	○	○
	Insomnia (Athens Insomnia Scale [AIS]) (C) (N)	○	○
Social isolation	Social isolation (Lubben Social Network Scale [LSNS-6]) (C)	○	○
Depressive symptoms	Depressive symptoms (Center for Epidemiologic Studies Depression scale [CES-D]) (C) (N)	○	○
The Great East Japan	Memory of the earthquake disaster (C)	○	○
Earthquake	Experience of damage and loss due to the earthquake (C)	○	
	Physical damage caused by the earthquake (C)		○
	Evacuation behavior (C)	○	○
	Participation in disaster drill (past) (C)	○	○
	Participation in disaster drill (current) (C)		○
For women	About menstruation (C)	○	○
	Pregnancy/Childbirth (C) (N)	○	○
Health checkup	Participation of health checkup (C)	○	
	Vaccination (influenza/pneumococcus) (C)		○
Quality of life	Quality of life (EuroQol 5 Dimension) (N)	○	○
Irritable bowel syndrome	Irritable bowel syndrome (ROME-II modular questionnaire) (C)	○	○
Autism-Spectrum	Autism-Spectrum Quotient (AQ) (C)	○	○

Eating habit	Eating habit (C)	○	○
Food intake	Food Frequency Questionnaire (FFQ) (C) (N)	○	○

C, categorical data; GEJE, Great East Japan Earthquake; N, numerical data; PTSD, Post-Traumatic Stress Disorder.

#### Blood and urine tests

The volume of blood collected during the second period survey was 36 mL. Of this, 7 mL was placed in an EDTA 2Na bottle, 7 mL in a sodium heparin bottle, and 9 mL each in two plain bottles. Additionally, 2 mL of blood was used for a complete blood count test and another 2 mL for a blood sugar or HbA1c test. Participants were requested to visit the center on the morning of the survey with a urine sample collected at home. Thereafter, they were requested to bring the urine to the sites or assessment centers. The measurement items are listed in Table 3. In the second period survey, the serum pepsinogen test included in the baseline survey was removed and thyroid-stimulating hormone and C-reactive protein (CRP) tests were added.

**Table 3.** Details of blood, urine, and other tests at baseline and/or follow-up for the TMM CommCohort study

Measurement	Measurement lists	Baseline	second period
Blood	N-terminal pro-B-type natriuretic peptide (NT-proBNP) Glycoalbumin (GA)	○	○
	Creatinine (CRE)	○	○
	Allergen-Specific immunoglobulin E (IgE) antibody test	○	○
	Helicobacter pylori antibody test	○	
	Serum pepsinogen (PG) test	○	
	Aspartate aminotransferase (AST)	○	○
	Alanine aminotransferase (ALT)	○	○
	Gamma-glutamyl transferase (GGT)	○	○
	Urea nitrogen (UN)	○	○
	Uric acid (UA)	○	○
	Glucose	○	○
	Triglycerides (TG)	○	○
	Total cholesterol (T-Cho)	○	○
	Low-density lipoprotein cholesterol (LDL-C)	○	○
	High-density lipoprotein cholesterol (HDL-C)	○	○
	Complete blood count (CBC)	○	○
	Hemoglobin A1c (HbA1c)	○	○
	Immunoglobulin E (IgE) test	○	
	Cystatin C	○	○
	Thyroid stimulating hormone (TSH)		○
	C-reactive protein (CRP)		○

Urine	Microalbumin	○	○
	Creatinine (CRE)	○	○
	Sodium	○	○
	Chloride	○	○
	Potassium	○	○

Others	Height	○	○
	Weight and body composition	○^b^	○
	Waist circumference	○	○
	Touchscreen questionnaire	○^b^	○
	Axial length	○^b^	○
	Intraocular pressure	○^b^	○
	Optical coherence tomography	○^b^	○
	Color retinal photography	○^b^	○
	Refraction and keratometry	○^b^	○
	Hearing acuity	○^b^	○
	Respiratory function	○^b^	○
	Respiratory impedance	○^b^	○
	Estimated central aortic blood pressure, casual blood pressure and heart rate	○^b^	○
	Carotid ultrasound imaging	○^b^	○
	Calcaneal ultrasound bone mineral density	○^b^	○
	Leg extension strength	○^b^	
	Grip strength	○^b^	○
	Oral examination	○^b^	○
	Oral bacteriological examination	○^b^	○
	Home blood pressure^a^	○^b^	○
	Number of steps per day^a^	○^b^	○
	Fraction of exhaled nitric oxide (FeNO)		○
	Electrocardiograph		○
	Taste of salt		○

^a^Measured for around 10 days.

^b^Measured only Type 1 with CSC survey or Type 2 survey participants at the baseline survey.

#### Physiological tests

Various physiological tests were conducted to evaluate the subjects’ current physical condition in detail (Table 3). In the second period survey, the leg extension strength test performed in the baseline survey was removed, the fraction of exhaled nitric oxide was assessed (Niox Vero; Chest M.I., Inc., Tokyo, Japan), and electrocardiogram (ECG-2500; Nihon Kohden Corporation, Tokyo, Japan) was performed.

#### Add-on tests

Various collaborative studies are being conducted in addition to the abovementioned tests. There is collaborative research with Yakult Central Institute on lacto-fermented beverages and intestinal flora, and another collaboration with OMRON Healthcare Co., Ltd to produce a urine sodium (Na)/potassium (K) ratio meter, an activity tracker, and a sleep meter.

#### Death information

Tracking of deceased participants was performed by browsing the basic resident registration of the local government in the following cases: when mail, such as follow-up questionnaires from the TMM CommCohort secretariat, was not delivered; when family members provided an individual’s information; or when information on death-related withdrawal from the National Health Insurance was provided. The basic resident registration was viewed at least once a year when an individual could not be contacted.

### Statistical analyses

The characteristics of the second period survey participants were examined according to the participation types in the baseline survey. For each participation type, we divided the group into those who participated in the second period survey and those who did not. Specifically, we examined age, sex, degree of house damage, social factors, including education, marital status, and LSNS-6; lifestyle factors, including smoking, alcoholism, and time spent walking; physical factors, including body mass index (BMI) and medical history; and psychological factors, including perceived stress, K6 score, and CES-D score.

We also examined the association between participation type and second period survey characteristics such as smoking (never-smoker, and ever-smoker), drinking (never-drinker, and ever-drinker), time spent walking (less than 1 hour, or 1 hour or more), BMI (less than 25 kg/m^2^, or 25 kg/m^2^ or more), medical history (presence or absence of cancer, stroke, or myocardial infarction), perceived stress (minimal or none, or perceived), psychological distress (K6 score; less than 13, or 13 or more), or depressive symptoms (CES-D score; less than 16, or 16 or more). Multivariate logistic regression was used to calculate odds ratios (ORs) and 95% confidence intervals (CIs) after controlling for potential confounders. The variables considered in the models were age at the second period survey (continuous), sex, education at baseline (16 years or more, 13 to 15 years, 12 years or less, or unknown), marital status at the second period survey (married, single, separated, widowed, or unknown), social isolation at the second period survey (LSNS-6 score; less than 12, 12 or more, or unknown), and situation of disaster damage at baseline (not living in the disaster area or no damage to the house, partial damage to the house, marked damage or destruction of the house, or unknown).

## RESULTS

**Figure 1.**
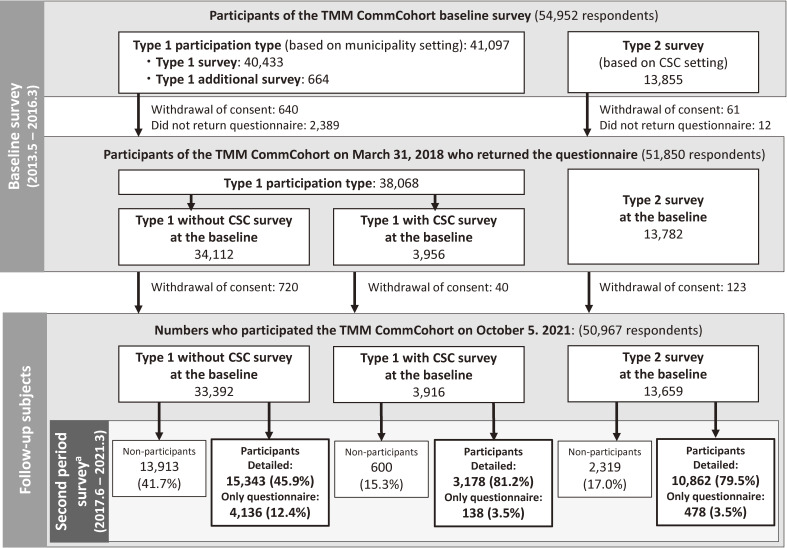
Flow chart of the Miyagi part of the TMM CommCohort Study from the baseline survey to second period survey. ^a^second period survey: the repeated center-based survey. CSC, Community Support Centers

Of 54,952 baseline survey participants, 50,967 continued to consent to the survey as of October 5, 2021. In addition, as of March 2021, 1,053 participants were confirmed dead based on public data. A total of 33,392 participants participated in the baseline Type 1 without CSC survey, and 3,916 participants participated in the baseline Type 1 with CSC survey; 15,343 (45.9%) of the former group and 3,178 (81.2%) of the latter group participated in the second period survey. Further, 10,862 (79.5%) of 13,659 Type 2 survey participants participated in the survey (Figure 1). Out of 50,967 participants, 29,383 (57.3%) participated in the second period survey in Miyagi Prefecture.

Among those who had difficulty visiting the CSC, whom we asked to return the filled questionnaire, 4,752 returned the questionnaire. With the addition of this group, 34,135 participants (67.0%) participated in the second period survey.

### Baseline characteristics of the participants according to whether they participated in the second period survey

Regarding the mean age of the participants at baseline, there was a tendency for Type 2 participants to be younger than the other participants. Particularly, Type 2 participants who did not participate in the second period survey were younger than the other participants (Table 4). Type 1 without CSC survey and Type 2 participants who participated in the second period survey were more likely to be married, 60–69 years old, and never-smokers, with fewer depressive symptoms, compared to those who did not participate. Furthermore, regardless of whether or not they participated in the second period survey, Type 1 with CSC survey participants were more likely to have completed at least 16 years of education. Type 2 survey participants were more likely to have experienced partial house damage during the disaster, and their BMI was less than 25 kg/m^2^ at baseline. There were not many differences in the other variables between those who did or did not participate in the second period survey.

**Table 4.** Baseline characteristics of participants according to whether or not they participated in the second period survey

	Total subjects at the baseline survey	Baseline survey participation type					

		Type 1 without CSC survey (*N* = 33,392)		Type 1 with CSC survey (*N* = 3,916)		Type 2 survey (*N* = 13,659)	

		second period survey					

		Not participated	Participated	Not participated	Participated	Not participated	Participated

	Number (%)	Number (%)	Number (%)	Number (%)	Number (%)	Number (%)	Number (%)
**Total participants**	50,967	18,049 (54.1)	15,343 (45.9)	738 (18.8)	3,178 (81.2)	2,797 (20.5)	10,862 (79.5)

Age at baseline, years							
Mean (SD)	59.0 (12.3)	58.9 (12.2)	60.2 (11.0)	60.3 (11.2)	60.6 (10.1)	55.1 (16.3)	57.9 (13.0)
20–39	5,236 (10.3)	1,966 (10.9)	1,211 (7.9)	55 (7.5)	169 (5.3)	627 (22.4)	1,208 (11.1)
40–49	5,471 (10.7)	1,874 (10.4)	1,329 (8.7)	79 (10.7)	297 (9.4)	417 (14.9)	1,475 (13.6)
50–59	8,878 (17.4)	3,007 (16.7)	2,481 (16.2)	110 (14.9)	578 (18.2)	430 (15.4)	2,272 (20.9)
60–69	21,941 (43.1)	7,739 (42.9)	7,562 (49.3)	352 (47.7)	1,594 (50.2)	707 (25.3)	3,987 (36.7)
≥70	9,441 (18.5)	3,463 (19.2)	2,760 (18.0)	142 (19.2)	540 (17.0)	616 (22.0)	1,920 (17.7)
Sex							
Male	18,281 (35.9)	7,267 (40.3)	5,753 (37.5)	220 (29.8)	957 (30.1)	912 (32.6)	3,172 (29.2)
Female	32,686 (64.1)	10,782 (59.7)	9,590 (62.5)	518 (70.2)	2,221 (69.9)	1,885 (67.4)	7,690 (70.8)
**Social factors**							
Education							
16 years or more	5,893 (11.6)	1,452 (8.0)	1,672 (10.9)	57 (7.7)	405 (12.7)	458 (16.4)	1,849 (17.0)
13–15 years	11,984 (23.5)	3,816 (21.1)	3,568 (23.3)	192 (26.0)	818 (25.7)	712 (25.5)	2,878 (26.5)
12 years or less	32,090 (63.0)	12,253 (67.9)	9,850 (64.2)	479 (64.9)	1,899 (59.8)	1,587 (56.7)	6,022 (55.4)
Unknown	1,000 (2.0)	528 (2.9)	253 (1.7)	10 (1.4)	56 (1.8)	40 (1.4)	113 (1.0)
Marital status							
Married	39,827 (78.1)	13,721 (76.0)	12,462 (81.2)	593 (80.4)	2,573 (81.0)	1,967 (70.3)	8,511 (78.4)
Single	4,067 (8.0)	1,619 (9.0)	951 (6.2)	36 (4.9)	213 (6.7)	379 (13.6)	869 (8.0)
Separate	2,339 (4.6)	787 (4.4)	635 (4.1)	45 (6.1)	132 (4.2)	148 (5.3)	592 (5.5)
Widowed	3,708 (7.3)	1,301 (7.2)	1,081 (7.1)	55 (7.5)	232 (7.3)	245 (8.8)	794 (7.3)
Unknown	1,026 (2.0)	621 (3.4)	214 (1.4)	9 (1.2)	28 (0.9)	58 (2.1)	96 (0.9)
Social isolation (LSNS-6 score)							
Socially isolated (Less than 12)	11,811 (23.2)	4,390 (24.3)	3,621 (23.6)	173 (23.4)	703 (22.1)	605 (21.6)	2,319 (21.4)
Socially integrated (12 or more)	36,765 (72.1)	12,456 (69.0)	11,129 (72.5)	529 (71.7)	2,371 (74.6)	2,048 (73.2)	8,232 (75.8)
Unknown	2,391 (4.7)	1,203 (6.7)	593 (3.9)	36 (4.9)	104 (3.3)	144 (5.2)	311 (2.9)
**Disaster damage situation**							
Not living in the disaster area ​ or No damage to the house	14,788 (29.0)	5,391 (29.9)	4,374 (28.5)	195 (26.4)	831 (26.2)	884 (31.6)	3,113 (28.7)
Partial damage to the house	27,931 (54.8)	9,657 (53.5)	8,863 (57.8)	394 (53.4)	1,802 (56.7)	1,338 (47.8)	5,877 (54.1)
Marked damage to the house ​ or Destruction of the house	5,773 (11.3)	1,837 (10.2)	1,452 (9.5)	115 (15.6)	408 (12.8)	438 (15.7)	1,523 (14.0)
Unknown	2,475 (4.9)	1,164 (6.5)	654 (4.3)	34 (4.6)	137 (4.3)	137 (4.9)	349 (3.2)
**Lifestyle factors**							
Smoking							
Never-smoker	30,851 (60.5)	10,168 (56.3)	9,366 (61.0)	461 (62.5)	2,144 (67.5)	1,669 (59.7)	7,043 (64.8)
Ex-smoker	12,349 (24.2)	4,130 (22.9)	3,953 (25.8)	159 (21.5)	756 (23.8)	627 (22.4)	2,724 (25.1)
Current smoker	6,816 (13.4)	3,138 (17.4)	1,786 (11.6)	99 (13.4)	247 (7.8)	483 (17.3)	1,063 (9.8)
Unknown	951 (1.9)	613 (3.4)	238 (1.6)	19 (2.6)	31 (1.0)	18 (0.6)	32 (0.3)
Drinking							
Never-drinker	22,445 (44.0)	8,205 (45.5)	6,673 (43.5)	340 (46.1)	1,476 (46.4)	1,169 (41.8)	4,582 (42.2)
Ex-drinker	1,310 (2.6)	511 (2.8)	365 (2.4)	25 (3.4)	71 (2.2)	82 (2.9)	256 (2.4)
Current drinker	26,870 (52.7)	9,097 (50.4)	8,224 (53.6)	367 (49.7)	1,620 (51.0)	1,541 (55.1)	6,021 (55.4)
Unknown	342 (0.7)	236 (1.3)	81 (0.5)	6 (0.8)	11 (0.4)	5 (0.2)	3 (0.03)
Time spent walking							
1 hour or more	26,729 (52.4)	9,575 (53.1)	8,201 (53.5)	402 (54.5)	1,719 (54.1)	1,389 (49.7)	5,443 (50.1)
Less than 1 hour	21,937 (43.0)	7,195 (39.9)	6,394 (41.7)	298 (40.4)	1,320 (41.5)	1,386 (49.6)	5,344 (49.2)
Unknown	2,301 (4.5)	1,279 (7.1)	748 (4.9)	38 (5.2)	139 (4.4)	22 (0.8)	75 (0.7)
**Physical factors**							
BMI							
Less than 25 kg/m^2^	36,830 (72.3)	12,436 (68.9)	11,197 (73.0)	514 (69.7)	2,307 (72.6)	1,996 (71.4)	8,380 (77.2)
25 kg/m^2^ or more	13,891 (27.3)	5,594 (31.0)	4,130 (26.9)	213 (28.9)	854 (26.9)	740 (26.5)	2,360 (21.7)
Unknown	246 (0.5)	19 (0.1)	16 (0.1)	11 (1.5)	17 (0.5)	61 (2.2)	122 (1.1)
Medical history							
Cancer	3,828 (7.5)	1,278 (7.1)	1,218 (7.9)	47 (6.4)	253 (8.0)	200 (7.2)	832 (7.7)
Stroke	1,101 (2.2)	414 (2.3)	323 (2.1)	18 (2.4)	81 (2.5)	56 (2.0)	209 (1.9)
Myocardial infarction, angina	1,409 (2.8)	498 (2.8)	421 (2.7)	21 (2.8)	97 (3.1)	86 (3.1)	286 (2.6)
**Psychological factors**							
Perceived stress							
Minimal or none	12,978 (25.5)	4,871 (27.0)	4,054 (26.4)	167 (22.6)	771 (24.3)	592 (21.2)	2,523 (23.2)
Significant or moderate	37,420 (73.4)	12,785 (70.8)	11,172 (72.8)	570 (77.2)	2,399 (75.5)	2,180 (77.9)	8,314 (76.5)
Unknown	569 (1.1)	393 (2.2)	117 (0.8)	1 (0.1)	8 (0.3)	25 (0.9)	25 (0.2)
Psychological distress (K6 score)							
Absence (Less than 13)	46,770 (91.8)	16,231 (89.9)	14,255 (92.9)	686 (93.0)	2,951 (92.9)	2,506 (89.6)	10,141 (93.4)
Presence (13 or more)	3,085 (6.1)	1,079 (6.0)	860 (5.6)	41 (5.6)	208 (6.5)	226 (8.1)	671 (6.2)
Unknown	1,112 (2.2)	739 (4.1)	228 (1.5)	11 (1.5)	19 (0.6)	65 (2.3)	50 (0.5)
Depressive symptoms (CES-D score)							
Absence (Less than 16)	36,098 (70.8)	12,200 (67.6)	11,118 (72.5)	522 (70.7)	2,280 (71.7)	1,881 (67.3)	8,097 (74.5)
Presence (16 or more)	12,388 (24.3)	4,491 (24.9)	3,620 (23.6)	176 (23.9)	814 (25.6)	770 (27.5)	2,517 (23.2)
Unknown	2,481 (4.9)	1,358 (7.5)	605 (3.9)	40 (5.4)	84 (2.6)	146 (5.2)	248 (2.3)

CSC, Community Support Centers (Established by ToMMo in seven areas in Miyagi Prefecture).

### Association between baseline survey participation type and the second period survey characteristics

**Figure 2.**
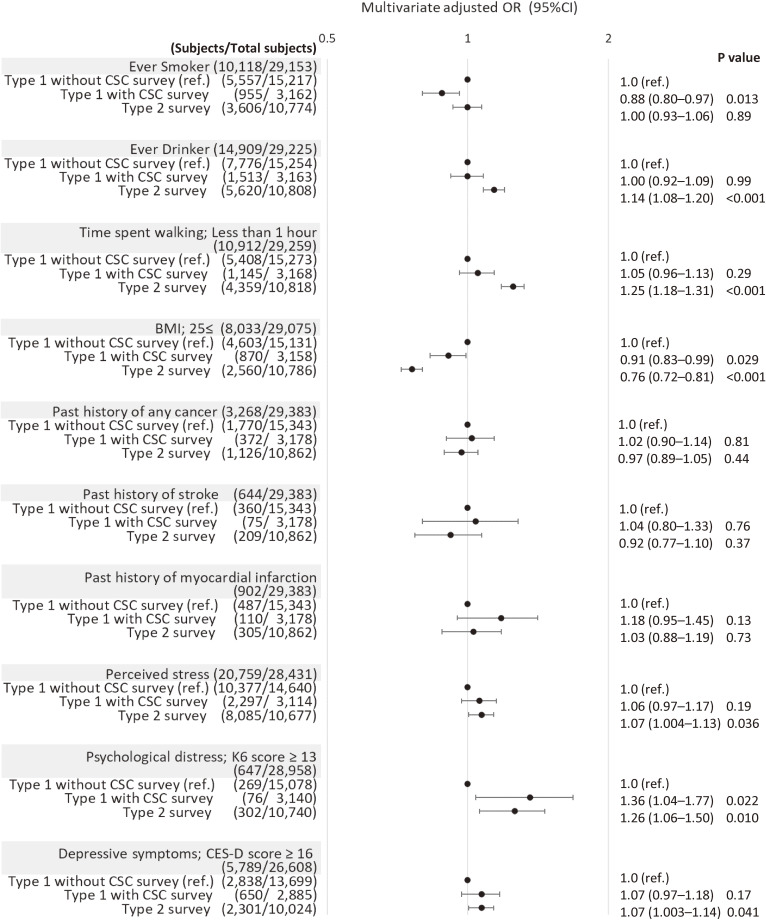
Multivariate adjusted odds ratio (OR) and 95% confidence interval (CI) for study participant characteristics at the second period survey, according to the baseline participation type. Multivariate adjusted OR controlled for age at the second period survey (continuous), sex, education at baseline (16 years or more, 13 to 15 years, 12 years or less, or unknown), marital status at the second period survey (married, single, separate, widowed, or unknown), social isolation at second period survey (LSNS-6 score: less than 12, 12 or more, or unknown), and disaster damage situation at baseline (not living in the disaster area or no damage to the house, partial damage to the house, marked damage to the house or destruction of the house, or unknown). Ever-smoker includes ex-smoker and current smoker. Ever-drinker includes ex-drinker and current drinker. BMI, body mass index; CES-D, Center for Epidemiologic Studies Depression Scale; CSC, Community Support Centers (Established by ToMMo in seven areas in Miyagi Prefecture); K6, Kessler Psychological Distress Scale; LSNS-6, Lubben Social Network Scale.

Figure 2 shows the ORs and 95% CIs for logistic regression analyses of lifestyle, physical, and psychological factors according to participation type, with adjustments for the abovementioned characteristics. Compared to Type 1 without CSC survey participants, Type 2 participants had higher odds of ever drinking (adjusted OR 1.14; 95% CI, 1.08–1.20) and less time spent walking (adjusted OR 1.25; 95% CI, 1.18–1.31) with lower odds of having a 25 kg/m^2^ or more BMI (adjusted OR 0.76; 95% CI, 0.72–0.81), and higher odds of perceived stress (adjusted OR 1.07; 95% CI, 1.004–1.13), psychological distress (adjusted OR 1.26; 95% CI, 1.06–1.50), and depressive symptoms (adjusted OR 1.07; 95% CI, 1.003–1.14). Additionally, Type 1 with CSC survey participants were associated with lower odds of an ever-smoker status (adjusted OR 0.88; 95% CI, 0.80–0.97), lower odds of having a 25 kg/m^2^ or more BMI (adjusted OR 0.91; 95% CI, 0.83–0.99), and higher odds of having psychological distress (adjusted OR 1.36; 95% CI, 1.04–1.77) compared to Type 1 without CSC survey participants.

## DISCUSSION

This paper provides an overview of the Miyagi Prefecture part of the second period survey of the TMM CommCohort Study. Nearly half of the 33,392 Type 1 without CSC survey participants in the baseline survey who visited only their municipalities’ specific health checkup venues also visited the regional support center and provided more detailed data. Furthermore, approximately 80% of the 17,575 participants who visited the CSC in the baseline survey subsequently attended the CSC during the second period survey. This allowed us to evaluate changes in health information, as shown in Table 2 and Table 3. We could also collect extensive data for approximately 30,000 participants in the second period survey.

### Characteristics of the second period survey participants

The second period survey participants may have had higher health consciousness and better health condition than the non-participants. Notably, these survey participants had a high percentage of never-smokers, individuals with a <25 kg/m^2^ BMI, and fewer depressive symptoms. Considering that the study included a healthy sample, the prevalence of diseases in real-life may have been underestimated; thus, caution is required in generalizing the study findings. However, participation rate was high, especially in participants who had visited CSC in baseline survey.

Type 1 with CSC survey and Type 2 survey respondents participated in a detailed survey by voluntarily visiting the CSC at baseline. Participants who voluntarily visited the CSC at the baseline survey may have been more interested in their health than the other participants, and they more actively participated in the second period survey. However, we hypothesized that Type 1 without CSC survey respondents might be health-conscious individuals receiving municipal-specific health checkups.^3^ When we examined whether lifestyle, physical, and psychological factors differed between the participation types after controlling for differences in sex, age, and social background, we found that Type 2 participants were more likely to consume alcoholic drinks, walk for less than an hour, and have a BMI of ≥25 kg/m^2^, perceived stress, psychological distress, and depressive symptoms. Although second period survey participants generally share the same characteristics, there are some variables that differ depending on the type of participation and need to be handled with care.

### Newly added variables in the second period survey

Some variables not considered in the baseline survey were added to the second period survey. For example, the questionnaire asked for information on, income, physical damage caused by the earthquake, participation in evacuation drills, *Helicobacter pylori* eradication status, vaccination behavior, and family care. C-reactive protein, fraction of exhaled nitric oxide, electrocardiogram, and taste of salt were added to the blood and urine tests in the baseline survey. The inclusion of these variables enabled a more detailed examination of the effects of the earthquake-related disaster and elucidation of issues, such as aging and infectious diseases, which have become public health concerns. The advantage of a face-to-face survey is that it allows the timely collection of required data, and we can proceed with a cohort study after this survey.

Furthermore, by establishing this cohort, many additional studies have become feasible, and industry-academia collaboration is steadily progressing. We are working on producing a Na/K ratio meter, an activity tracker, and a sleep meter with OMRON Healthcare Co., Ltd., as well as researching lacto-fermented beverage consumption and intestinal flora with Yakult Central Institute.

The strength of our study is that this cohort is one of Japan’s largest to be included in a prospective study, with extensive evaluations that are not commonly performed at regular health checkups. There are larger prospective cohorts with extensive evaluations, such as the Hisayama study^18^^,^^19^ and the Nagahama study.^20^ We performed 14,000 extensive evaluations (ie, there were 14,000 participants who visited the CSC at both baseline and second period surveys). These numbers were comparable to the aforementioned studies. Furthermore, the second period survey alone resulted in the creation of a massive database of approximately 30,000 people. The study information can be used to establish a database for observing changes in test values in conjunction with baseline surveys, as well as a large database for the next repeated center-based survey during the third period. Databases containing information on motor, visual, and auditory functions are particularly valuable in an aging society.

As with other prospective studies, our study has limitations. The participation ratio in the second period survey was approximately 60%. It is possible that these participants were healthier or more interested than non-participants. However, approximately 80% of individuals who visited CSC for the baseline survey attended the subsequent second period survey. Furthermore, despite not participating in the second period survey, 50,967 (98%) of the 51,850 individuals who completed the baseline survey continued to participate in the follow-up survey (ie, mail survey and survey based on public information, indicating a remarkably high follow-up rate).

### Conclusion

Following up the participants elucidated the long-term effects of disaster-related factors on health. By examining data from repeated measures, it is expected that future studies will be able to clarify the association between changes in survey items and the onset of disease. We also have genomic and other omics data,^1^^,^^21^^,^^22^ and by using this cohort, we could clarify the long-term effects of earthquake-related disaster and contribute to illness prevention and individualized treatment. Furthermore, we promote the use of these data in collaborative research.
